# Awareness, Access and Use of Shear Wave Elastography and MyotonPRO Among Physiotherapy Students and Practicing Physiotherapists: A Cross-Sectional Survey

**DOI:** 10.3390/healthcare14203440

**Published:** 2026-10-14

**Authors:** Sebastian Szajkowski, Jarosław Pasek, Grzegorz Cieślar

**Affiliations:** 1 Faculty of Medical Sciences, Warsaw Medical Academy of Applied Sciences, 8 Rydygiera St., 01-793 Warszawa, Poland; sebastianszajkowski@wp.pl; 2 Collegium Medicum, Jan Długosz University in Częstochowa, 13/15 Armii Krajowej St., 42-200 Częstochowa, Poland; 3 Department of Internal Medicine, Angiology and Physical Medicine, Faculty of Medical Sciences in Zabrze, Medical University of Silesia in Katowice, 15 Stefana Batorego St., 41-902 Bytom, Poland; cieslar1@o2.pl

**Keywords:** education, elasticity imaging techniques, musculoskeletal physiological phenomena, physical therapists, physical therapy specialty, professional

## Abstract

**Background:** Shear wave elastography (SWE) and MyotonPro provide quantitative information on tissue mechanical properties and may complement conventional musculoskeletal assessment. This study assessed awareness, access, use, and perceived clinical utility of these technologies among physiotherapy students and practicing physiotherapists, and examined associations with age and professional experience. **Methods:** A cross-sectional observational study was conducted using an anonymous online survey. The author-developed questionnaire assessed awareness, access, self-reported use, self-rated knowledge, perceived utility, training, and implementation barriers. The sample included 101 respondents: 45 final-year physiotherapy students and 56 practicing physiotherapists from Warsaw, Poland. Group differences and associations with age and professional experience were assessed using appropriate non-parametric and categorical tests, with Holm adjustment for multiple comparisons. **Results:** Awareness of SWE and MyotonPro was reported by 31.7% and 35.6% of respondents, respectively, while 29.7% had access to either technology. Ever-use was reported by 15.8% for SWE and 21.8% for MyotonPro. Practicing physiotherapists showed greater awareness and use than students (SWE: 51.8% vs. 6.7%; MyotonPro: 53.6% vs. 13.3%; both *p* < 0.001). All nine group comparisons remained significant after adjustment. Professional experience was positively associated with all nine outcomes (ρ = 0.30–0.62; all adjusted *p* ≤ 0.0032), with the strongest association for SWE self-rated knowledge (ρ = 0.62, adjusted *p* < 0.0001). Age showed weaker associations. **Conclusions:** SWE and MyotonPro remain incompletely integrated into the surveyed physiotherapy population. Professional experience was the most consistent correlate of technology-related outcomes. Limited equipment access and cost were important barriers. The findings highlight differences in awareness, experience, and perceived barriers, while further research is needed to determine whether educational or organizational interventions would improve clinical adoption.

## 1. Introduction

Quantitative assessment of tissue mechanical properties has gained increasing interest as a potential complement to conventional musculoskeletal assessment in physiotherapy [1,2]. Such technologies may provide quantitative information on tissue characteristics that are not directly captured by routine clinical examination or conventional structural imaging. These approaches may be particularly relevant in conditions involving progressive or load-related tissue changes, including overuse-related disorders [3]. However, the present study does not assess the diagnosis, monitoring, or treatment of overuse injuries. Rather, it focuses on the broader implementation context of quantitative tissue-assessment technologies by examining awareness, access, use, perceived clinical utility, and implementation-related factors among physiotherapy students and practicing physiotherapists.

Conventional imaging, including radiography, B-mode ultrasonography, and magnetic resonance imaging (MRI), remains central to musculoskeletal assessment because of its established role in detecting structural abnormalities. However, structural findings may not fully correspond to symptoms, functional impairment, or tissue mechanical behavior. Quantitative assessment of tissue mechanical properties may therefore provide complementary information to conventional clinical assessment [1]. Shear wave elastography (SWE) provides quantitative information related to tissue stiffness by assessing shear-wave propagation and has been investigated in skeletal muscle and tendon [2]. However, its clinical interpretation depends on standardized measurement procedures, and substantial methodological heterogeneity has been reported across SWE studies, including differences in measurement protocols, anatomical positioning, and technical parameters. A recent systematic review of 42 studies of rotator cuff muscle SWE found moderate evidence for reliability but inconsistent evidence for convergent validity, discriminant validity, and responsiveness, and emphasized the need for improved standardization of measurement procedures [4]. MyotonPro (Myoton AS, Tallinn, Estonia) is a portable, non-invasive device that applies a brief mechanical impulse to the skin overlying a selected tissue and records the resulting tissue response to quantify mechanical properties such as stiffness, tone, and elasticity. It has demonstrated promising reliability across a range of muscles, although methodological heterogeneity and limitations in the evidence for specific parameters remain [5,6].

The potential clinical use of these technologies also depends on appropriate education and practitioner competence. Previous research concerning SWE has predominantly examined its measurement properties, methodological standardization, and potential clinical applications rather than awareness or educational exposure among physiotherapists. SWE studies in musculoskeletal tissues have demonstrated substantial methodological heterogeneity, including differences in ultrasound systems, transducers, measurement depth, tissue position, and acquisition procedures, which limits direct comparison and generalization of findings [4,7]. A survey of musculoskeletal radiologists also indicated that, although SWE was available in some institutions, clinical use remained limited, illustrating the gap between technological availability and routine clinical implementation [8]. However, this evidence comes from a different professional population and cannot be directly extrapolated to physiotherapists.

The literature concerning MyotonPro has similarly focused mainly on reliability, validity, feasibility, and potential clinical applications rather than awareness or education among physiotherapists. Systematic reviews have reported generally favorable reliability for several MyotonPro parameters, but have also identified methodological heterogeneity and limited evidence for particular parameters, anatomical sites, and clinical applications [5,6]. Clinical feasibility studies have additionally examined the use of MyotonPro in physiotherapy settings and have reported that the technique is feasible for clinical measurement, while also highlighting practical considerations related to measurement procedures and interpretation [9]. These studies provide information about the feasibility and measurement properties of MyotonPro but do not establish how familiar physiotherapists are with the technology or how frequently it is used in routine practice.

Evidence from general ultrasound research provides relevant contextual information but cannot be assumed to apply directly to SWE or MyotonPro. International surveys of physiotherapists have identified training, equipment availability, cost, and uncertainty regarding scope of practice as important factors affecting ultrasound imaging use [10,11,12]. Broader research on technology use and standardized measurement instruments in physiotherapy has also identified implementation-related factors that may influence adoption of measurement technologies [13]. However, conventional ultrasound imaging and SWE are not interchangeable technologies: SWE adds quantitative tissue-mechanical assessment to ultrasound imaging and is influenced by tissue-specific and protocol-dependent measurement factors. MyotonPro differs further because it is a surface-applied mechanical impulse device rather than an ultrasound-based imaging technique. Its measurements are obtained from tissues accessible through the skin and are therefore primarily applicable to superficial tissues, whereas SWE can be used to assess tissue properties at greater depths depending on the ultrasound system, transducer, tissue, and measurement protocol. These differences in tissue accessibility, measurement procedures, and training requirements are important when considering the potential clinical applications of the two technologies. Therefore, findings concerning general ultrasound education or implementation should be interpreted as contextual evidence rather than direct evidence concerning adoption of SWE or MyotonPro.

Despite increasing research on the measurement properties and potential clinical applications of SWE and MyotonPro, limited evidence is available regarding their awareness, access, previous use, self-rated knowledge, perceived clinical utility, and implementation-related factors among physiotherapy students and practicing physiotherapists. The present study was designed to address this gap by examining these factors for both technologies and by comparing respondents at the end of undergraduate physiotherapy education with physiotherapists already working in clinical practice. This approach was intended to characterize the current implementation and educational context rather than to establish the clinical effectiveness of either technology. Final-year physiotherapy students were selected as a relevant comparison group because they are approaching entry into professional practice and therefore provide an opportunity to assess the level of awareness and perceived knowledge present at the end of undergraduate education. The present study did not assume that SWE or MyotonPro constitute established entry-level competencies in undergraduate physiotherapy education. Rather, the comparison was intended to examine whether technology-related awareness, perceived knowledge, use, and clinical perceptions differed between respondents at the end of undergraduate education and those already working in clinical practice. We hypothesized that greater professional experience would be associated with higher self-rated knowledge, previous use, and perceived clinical utility of SWE and MyotonPro.

Therefore, the primary aim of this study was to assess awareness, access, self-reported use, and perceived clinical utility of SWE and MyotonPro among physiotherapy students and practicing physiotherapists. The secondary aims were to examine differences between students and practicing physiotherapists and to determine whether age and professional experience were associated with self-rated knowledge, use, and perceptions.

## 2. Material and Methods

### 2.1. Study Design and Setting

A cross-sectional, quantitative observational study was conducted using an anonymous, self-administered online survey. The study was designed to assess awareness, access, self-reported use, and perceived clinical utility of SWE and MyotonPro among physiotherapy students and practicing physiotherapists, and to identify professional and sociodemographic factors associated with technology-related knowledge, attitudes, and adoption. The study was conducted in Warsaw, Poland. Participants were recruited from the local physiotherapy student and practicing physiotherapist population. Final-year students were enrolled in physiotherapy programs at different higher-education institutions in Warsaw, while practicing physiotherapists were employed in different clinical or professional settings across Warsaw. Thus, neither the student group nor the practicing physiotherapist group was restricted to a single institution or workplace. The survey was administered electronically using an online questionnaire. Participation was voluntary, anonymous, and unpaid. Participants were informed about the purpose of the study and the conditions of participation before completing the questionnaire. This study was reported in accordance with the Strengthening the Reporting of Observational Studies in Epidemiology (STROBE) guidelines for cross-sectional studies.

### 2.2. Participants and Eligibility Criteria

**Figure 1:**
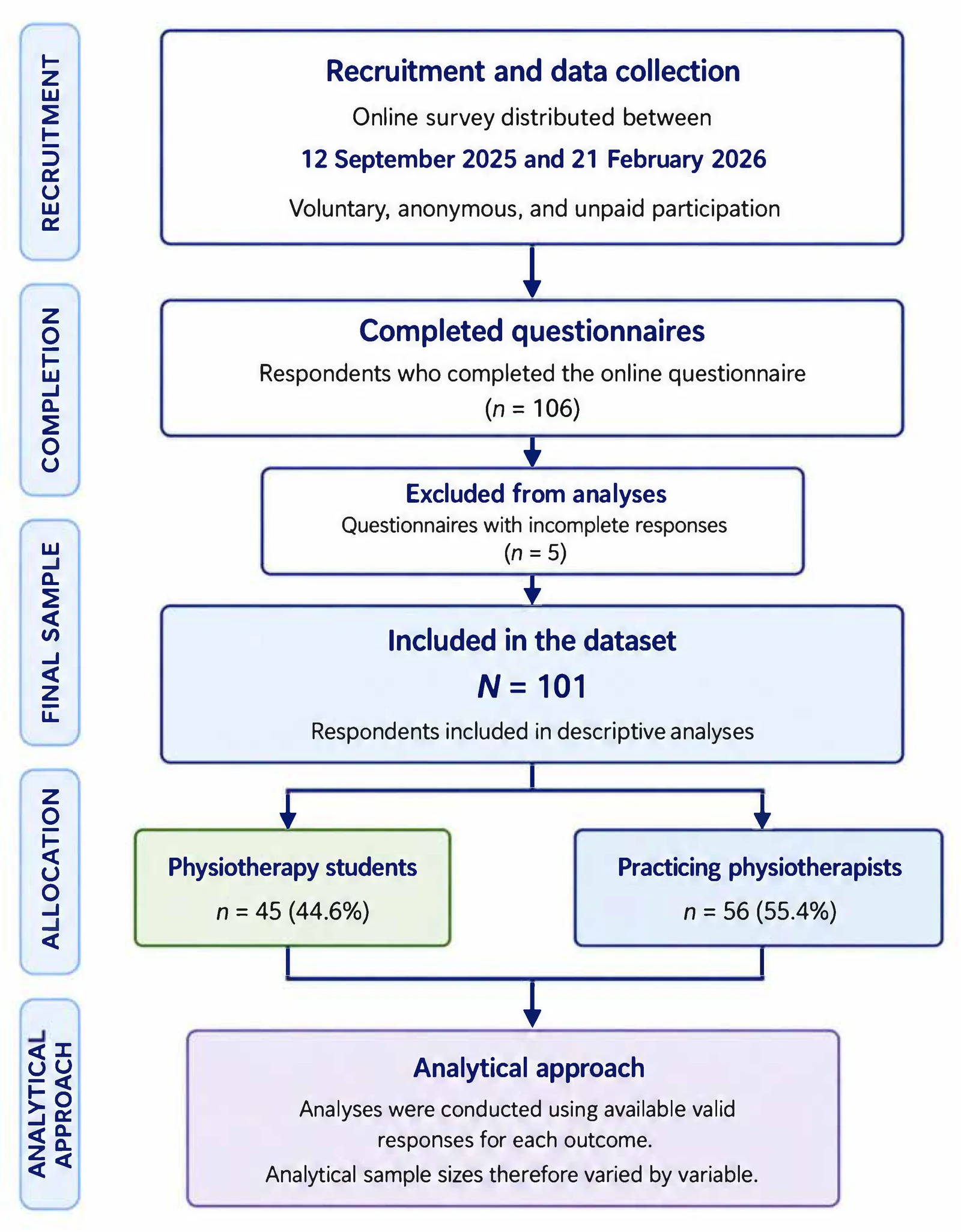
Participant recruitment, group allocation, and analytical approach.

Participants were recruited using purposive, voluntary sampling. Eligibility criteria were: (1) current enrollment in the final year of a physiotherapy programme or (2) active professional practice as a physiotherapist, and (3) voluntary agreement to participate. Eligibility for final-year student status and active professional practice was based on participants’ reported educational and professional status; no independent institutional or employment verification was performed. All student participants were studying in Warsaw, Poland, and all practicing physiotherapists were working in Warsaw, Poland. The participant recruitment process is shown in Figure 1.

### 2.3. Survey Instrument

Data were collected using an author-developed questionnaire specifically designed for this study. The items were generated by the authors based on the study objectives and the predefined domains of the survey, with the aim of assessing awareness, self-rated knowledge, access, previous use, perceived clinical utility, attitudes, training needs, and implementation barriers related to SWE and MyotonPro. The questionnaire was developed specifically for the present study and was not adapted from a previously validated questionnaire. No formal expert-panel review, pilot testing, cognitive interviewing, or formal assessment of item comprehensibility was conducted before the questionnaire was administered. The selection of questionnaire domains was informed by previous surveys of ultrasound use and education among physiotherapists, particularly studies addressing clinical use, training, knowledge, equipment access, and implementation barriers [10,11,12], as well as by literature concerning the measurement properties and potential clinical applications of SWE and MyotonPro [4,5,6]. The instrument comprised 32 substantive items and an automatically generated response timestamp field. The questionnaire covered four principal domains: (1) sociodemographic and professional characteristics; (2) previous awareness, access to, and use of SWE and MyotonPro; (3) knowledge, perceived clinical utility, and attitudes toward these technologies; and (4) barriers to implementation and potential clinical applications. The sociodemographic and professional section assessed age, educational level, workplace location, sex, professional experience, and main workplace. Respondents were additionally asked whether they had encountered patients presenting with symptoms suggestive of tissue overload despite the absence of abnormalities on conventional imaging (radiography, MRI, or B-mode ultrasonography), and how frequently they considered such imaging findings when making therapeutic decisions. Technology-related items assessed previous awareness of SWE and MyotonPro, access to either technology, self-rated knowledge of their operating principles, previous use, frequency of use, and clinical or research indications. The questionnaire also assessed perceptions regarding the ability of SWE and MyotonPro to identify early changes not apparent on conventional imaging, the perceived added value of combining both technologies, willingness to participate in training, perceived equipment- and cost-related barriers, and perceived usefulness for treatment monitoring. Two self-rated knowledge items were assessed using 0–5 scales. Attitudinal, perceived-utility, training, and barrier items were assessed using five-point ordinal scales. No substantive introductory information about the operating principles or clinical performance of SWE or MyotonPro was provided before the technology-related perception items. Participants were therefore asked to respond based on their pre-existing knowledge and experience. The questionnaire did not include a separate ‘unable to judge’ or ‘do not know’ response option for these ordinal perception items. The questionnaire also included questions regarding the most useful MyotonPro parameters, the most informative SWE findings, whether findings should be confirmed using another method, perceived barriers and benefits, and methodological suggestions. Questions concerning sources of information and selected clinical applications allowed respondents to select more than one answer. For these items, percentages for individual response categories were calculated using the number of respondents who provided a valid response to the respective question as the denominator. Because multiple responses could be selected, the sum of percentages could exceed 100%. For individual variables and analyses, available valid responses were used; consequently, the number of observations could vary between analyses. The complete reconstructed questionnaire, including the wording of the items, response options, and numerical rating scales, is provided as Supplementary Material S1.

### 2.4. Operationalization of Study Outcomes

The primary outcomes were awareness of SWE and MyotonPro, access to either technology, self-reported use, and perceived clinical utility. Secondary outcomes included self-rated knowledge, beliefs concerning potential early-change detection, perceived additional value of combining SWE and MyotonPro, willingness to undertake training, and perceived implementation barriers. Professional experience and age were treated as ordered categorical variables in the correlation analyses. Educational level and workplace location were recorded as additional descriptive variables. For the main group comparison, respondents were categorized as students or practicing physiotherapists according to the main workplace variable. Because the questionnaire was developed specifically for the present study, the available documentation supports describing it as an author-developed survey instrument. No formal psychometric validation, external validation, test–retest reliability assessment, or internal consistency analysis is documented in the supplied materials; therefore, no such validation properties are claimed. Accordingly, these variables were treated as study-specific self-report indicators rather than validated psychometric measures of latent constructs. The analyses therefore describe respondents’ reported knowledge, attitudes, and perceived clinical utility within this sample and do not establish the reliability or construct validity of these measures. Because item interpretation and measurement equivalence between students and practicing physiotherapists were not formally assessed, differences in how respondents understood or rated some items cannot be excluded.

### 2.5. Data Collection and Data Management

The questionnaire was distributed online via Facebook and Instagram, as well as by email. The survey link was shared through physiotherapy-related social media groups and professional networks, targeting physiotherapy students and practicing physiotherapists in Warsaw. The survey link was distributed through physiotherapy-related online communities and professional networks rather than being sent to a predefined list of individually identified participants. Participation was voluntary, and the questionnaire was not restricted to members of a single institution or professional organization. The specific number of groups, their membership size, and the number of individuals who received the invitation were not recorded; therefore, a response rate could not be calculated. Because the survey was anonymous and did not collect personally identifying information, duplicate responses could not be definitively excluded on the basis of participant identity. Responses were exported to a structured spreadsheet for statistical analysis. A total of 106 questionnaire responses were recorded during the data-collection period. Five responses were excluded from the final dataset because the corresponding questionnaires were incomplete, resulting in 101 respondents included in the descriptive and subsequent analyses. Within the retained dataset, item-level missing responses were present for some questionnaire items; these were retained as missing and were not imputed. Analyses were performed using the available valid observations for each variable, and therefore analytical sample sizes could differ between outcomes. The dataset contained response timestamps used to establish the data-collection period. No personally identifying variables were included in the analytical dataset supplied for this study. The study materials describe participation as anonymous and unpaid.

### 2.6. Ethical Considerations

The study was approved by the Bioethics Committee of the Warsaw Medical Academy of Applied Sciences, Warsaw, Poland (approval No. 202506WAM04; approval date: 30 June 2025). All participants provided informed consent before completing the anonymous online questionnaire. Participation was voluntary and unpaid, and participants were informed about the purpose of the study and the conditions of participation.

### 2.7. Statistical Analysis

Statistical analyses were performed using PQStat software (version 1.8.6). Categorical variables were summarized using absolute frequencies (n) and percentages (%). For ordinal and numeric variables, descriptive statistics included the mean (M), standard deviation (SD), median (Mdn), interquartile range (IQR), minimum, and maximum, as appropriate. Because the principal questionnaire outcomes were ordinal and demonstrated non-normal distributions, non-parametric methods were used for the primary inferential analyses. Differences between final-year physiotherapy students and practicing physiotherapists in ordinal self-rated knowledge, attitude, training, barrier, and perceived-utility outcomes were assessed using the two-sided Mann–Whitney U test. Results were summarized using the U statistic and two-sided *p* values. Rank-biserial correlation (r_rb) was calculated as a non-parametric effect size measure for the Mann-Whitney U comparisons. Associations between categorical variables, including professional status, previous awareness or use, and selected clinical-experience variables, were examined using Pearson’s chi-square test. Where appropriate, the phi coefficient (φ) was used to quantify the strength of association. Associations between technology-related outcomes and age or professional experience were assessed using Spearman’s rank correlation coefficient (ρ). Because multiple related outcomes were evaluated, Holm’s step-down procedure was applied to control the family-wise error rate within predefined families of related comparisons. Three multiplicity-adjusted families were considered: the nine student-versus-practicing-physiotherapist comparisons, the nine age correlations, and the nine professional-experience correlations. All statistical tests were two-sided, and statistical significance was defined a priori as α = 0.05. Exact *p* values were reported when available; values below 0.0001 were reported as *p* < 0.0001. No multivariable causal or predictive modelling was performed. The analyses were limited to prespecified bivariate group comparisons and correlation analyses. The analyses were intended to characterize associations between professional status, age, professional experience, and self-reported awareness, use, and perceptions of SWE and MyotonPro. Because the study was cross-sectional and survey-based, the analyses do not permit conclusions regarding diagnostic accuracy, prognostic performance, or clinical effectiveness of either technology.

Sample size considerations. Sample size adequacy was assessed using prior awareness of SWE as a representative primary outcome, based on the observed proportions of 6.7% among physiotherapy students (3/45) and 51.8% among practicing physiotherapists (29/56). The corresponding standardized effect size for the difference in proportions was large (Cohen’s h = 1.08). For a two-sided test with α = 0.05 and 80% power, approximately 28 participants in total would be required assuming the observed 45:56 group allocation. The final sample of 101 respondents therefore exceeded the estimated sample required to detect an effect of the observed magnitude with 80% power. This calculation was performed as a sample-size adequacy assessment based on the observed effect and should not be interpreted as an a priori sample-size determination. Because participation was voluntary, the final sample was determined by the number of eligible respondents completing the survey during the predefined data-collection period. Accordingly, the study should be considered exploratory with respect to the detection of associations and between-group differences, particularly for smaller effects and subgroup analyses.

## 3. Results

A total of 106 questionnaire responses were recorded during the data-collection period. Five incomplete questionnaires were excluded, resulting in 101 respondents included in the final analyses. For the principal comparative analyses, participants were classified according to their declared main occupational setting: 45 final-year physiotherapy students (44.6%) and 56 practicing physiotherapists (55.4%). The sample included 62 men (61.4%) and 39 women (38.6%). Age, educational level, workplace location, professional experience, and main workplace were recorded as participant characteristics. The occupational grouping was based on the declared main workplace item in the questionnaire. Educational level and professional experience were recorded as separate participant characteristics and were not used to define the study groups. The sociodemographic and professional characteristics of the participants are presented separately according to study group in Table 1. Students were predominantly younger and had less professional experience, whereas practicing physiotherapists were more frequently represented in the older age categories and had greater professional experience. Sex distribution was relatively similar between the groups.

**Figure 2:**
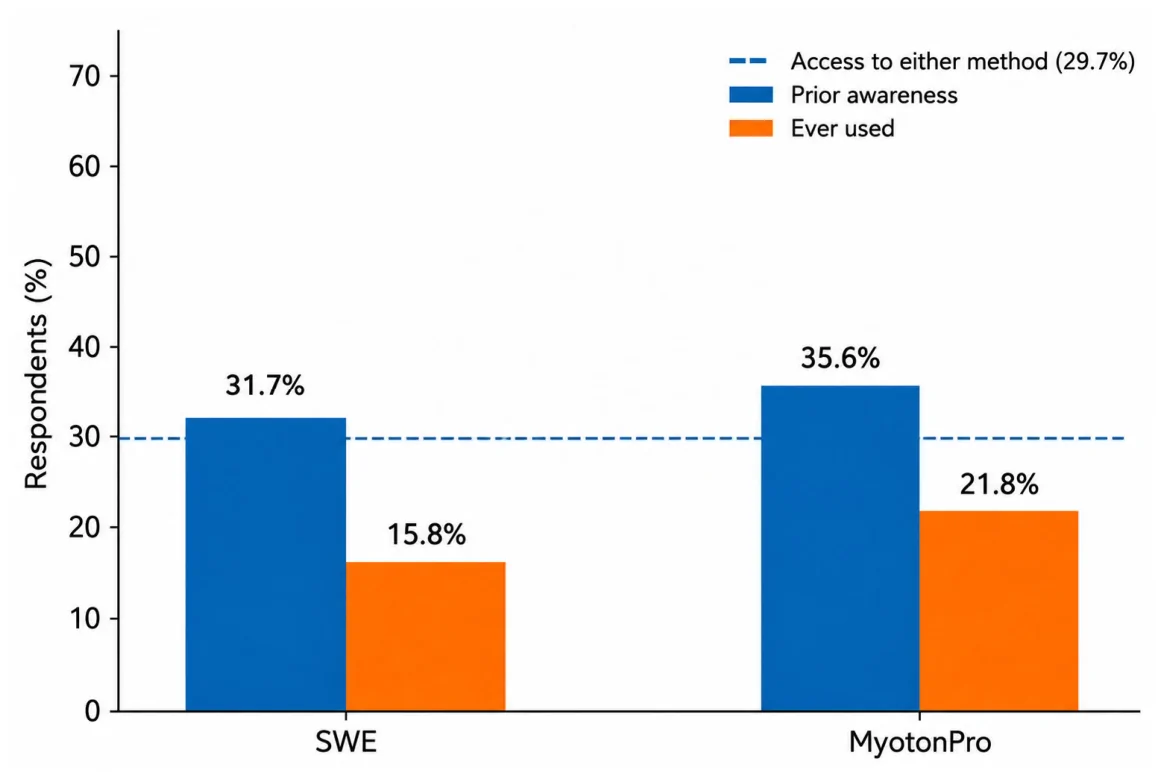
Overall awareness, access and self-reported use of SWE and MyotonPro (n = 101).

Overall, prior awareness of SWE was reported by 32/101 (31.7%) respondents and prior awareness of MyotonPro by 36/101 (35.6%). Only 30/101 (29.7%) respondents reported access to SWE and/or MyotonPro. Ever-use was reported by 16/101 (15.8%) respondents for SWE and 22/101 (21.8%) for MyotonPro, whereas regular use was uncommon, with one respondent reporting regular SWE use and four reporting regular MyotonPro use. The overall distribution of awareness, access, and self-reported use is illustrated in Figure 2.

Professional status was strongly associated with awareness and adoption of both technologies. Detailed between-group frequencies and corresponding chi-square statistics are presented in Table 2.

Practicing physiotherapists were substantially more likely than students to have previously heard of SWE (51.8% vs. 6.7%; χ^2^ = 23.47, *p* < 0.001, φ = 0.48) and MyotonPro (53.6% vs. 13.3%; χ^2^ = 17.61, *p* < 0.001, φ = 0.42). Access to at least one technology was also more common among practicing physiotherapists than students (46.4% vs. 8.9%; χ^2^ = 16.84, *p* < 0.001, φ = 0.41). No student reported having used SWE, whereas 28.6% of practicing physiotherapists reported previous use. For MyotonPro, ever-use was reported by 4.4% of students and 35.7% of practicing physiotherapists. Thus, all five categorical comparisons presented in Table 2 showed statistically significant differences between the two professional groups.

Self-rated knowledge was low in the full sample. The median score for self-rated knowledge of the operating principle was 0 for both SWE and MyotonPro. The median scores for perceived ability of SWE and MyotonPro to identify early changes were both 1 on the 1–5 response scales. However, exploratory analyses showed that these low overall scores were strongly related to prior awareness of the respective technologies. Among respondents who had previously heard of SWE, the median SWE early-change detection score was 3 (IQR 2.75–4), compared with 1 (IQR 1–1) among those without prior awareness. Similarly, the median MyotonPro early-change detection score was 3 (IQR 3–4) among respondents with prior awareness and 1 (IQR 1–1) among those without prior awareness. Among respondents reporting previous use, perceived utility was also higher, with a median of 4 (IQR 3–4) for SWE monitoring and 3 (IQR 3–4) for MyotonPro therapy utility, compared with medians of 1 (IQR 1–3) and 1 (IQR 1–2), respectively, among respondents without previous use. These exploratory findings suggest that the lowest response category may partly reflect limited familiarity with the technologies rather than uniformly unfavorable perceptions. Detailed exploratory analyses are provided in Supplementary Table S1.

Between-group comparisons of technology-related knowledge, perceptions, training willingness, barriers, and perceived clinical utility are presented in Table 3.

Practicing physiotherapists consistently reported higher scores than students across all nine technology-related outcomes. For SWE self-rated knowledge, the median score was 2.00 [0.00–4.00] among practicing physiotherapists compared with 0.00 [0.00–0.00] among students (U = 452.0, Holm-adjusted *p* < 0.0001, r_rb = 0.618). For MyotonPro self-rated knowledge, the corresponding medians were 2.00 [0.00–4.00] and 0.00 [0.00–0.00], respectively (U = 605.0, Holm-adjusted *p* < 0.0001, r_rb = 0.509). Practicing physiotherapists also reported stronger beliefs regarding the potential of SWE to identify early tissue changes (median 3.00 [1.00–4.00] vs. 1.00 [1.00–1.00]; U = 519.5, Holm-adjusted *p* < 0.0001, r_rb = 0.513) and MyotonPro (3.00 [1.00–3.00] vs. 1.00 [1.00–1.00]; U = 642.5, Holm-adjusted *p* = 0.0006, r_rb = 0.423). Perceived combined diagnostic value was also higher among practicing physiotherapists (median 3.00 [1.00–3.00] vs. 1.00 [1.00–1.00]; U = 698.5, Holm-adjusted *p* = 0.0022, r_rb = 0.369). Differences were also observed for training willingness, perceived access/cost barriers, and perceived clinical utility. Practicing physiotherapists reported greater willingness to undertake training (median 4.00 [2.00–4.75] vs. 1.50 [1.00–4.00]; U = 842.0, Holm-adjusted *p* = 0.0254, r_rb = 0.257) and stronger recognition of access and cost barriers (4.00 [1.00–5.00] vs. 1.00 [1.00–3.00]; U = 640.0, Holm-adjusted *p* = 0.0011, r_rb = 0.422). Perceived MyotonPro utility for therapy was higher among practicing physiotherapists (3.00 [1.00–3.00] vs. 1.00 [1.00–2.00]; U = 659.0, Holm-adjusted *p* = 0.0014, r_rb = 0.397), as was perceived SWE utility for treatment monitoring (3.00 [1.00–3.00] vs. 1.00 [1.00–1.00]; U = 667.5, Holm-adjusted *p* = 0.0022, r_rb = 0.374). All nine between-group comparisons remained statistically significant after Holm adjustment, with adjusted *p* values ranging from 0.0006 to 0.0254 (Table 3).

The largest rank-biserial effect sizes were observed for self-rated knowledge of SWE (r_rb = 0.618) and MyotonPro (r_rb = 0.509), followed by beliefs regarding the potential of SWE to identify early changes (r_rb = 0.513). Effect sizes were smaller for MyotonPro early-change beliefs (r_rb = 0.423), access/cost barriers (r_rb = 0.422), MyotonPro therapy utility (r_rb = 0.397), SWE monitoring utility (r_rb = 0.374), perceived combined diagnostic value (r_rb = 0.369), and training willingness (r_rb = 0.257).

Associations between age, professional experience, and the nine technology-related outcomes are summarized in Table 4.

Professional experience showed positive associations with all nine outcomes, with correlation coefficients ranging from ρ = 0.30 to 0.62. All nine associations remained statistically significant after Holm adjustment (all adjusted *p* ≤ 0.0032). The strongest association was observed between professional experience and SWE self-rated knowledge (ρ = 0.62, adjusted *p* < 0.0001). Professional experience was also positively associated with MyotonPro self-rated knowledge (ρ = 0.42, adjusted *p* < 0.0001), SWE early-change beliefs (ρ = 0.48, adjusted *p* < 0.0001), MyotonPro early-change beliefs (ρ = 0.46, adjusted *p* < 0.0001), perceived combined diagnostic value (ρ = 0.42, adjusted *p* = 0.0001), training willingness (ρ = 0.30, adjusted *p* = 0.0032), access/cost barriers (ρ = 0.47, adjusted *p* < 0.0001), MyotonPro therapy utility (ρ = 0.35, adjusted *p* = 0.0013), and SWE monitoring utility (ρ = 0.42, adjusted *p* = 0.0001).

**Figure 3:**
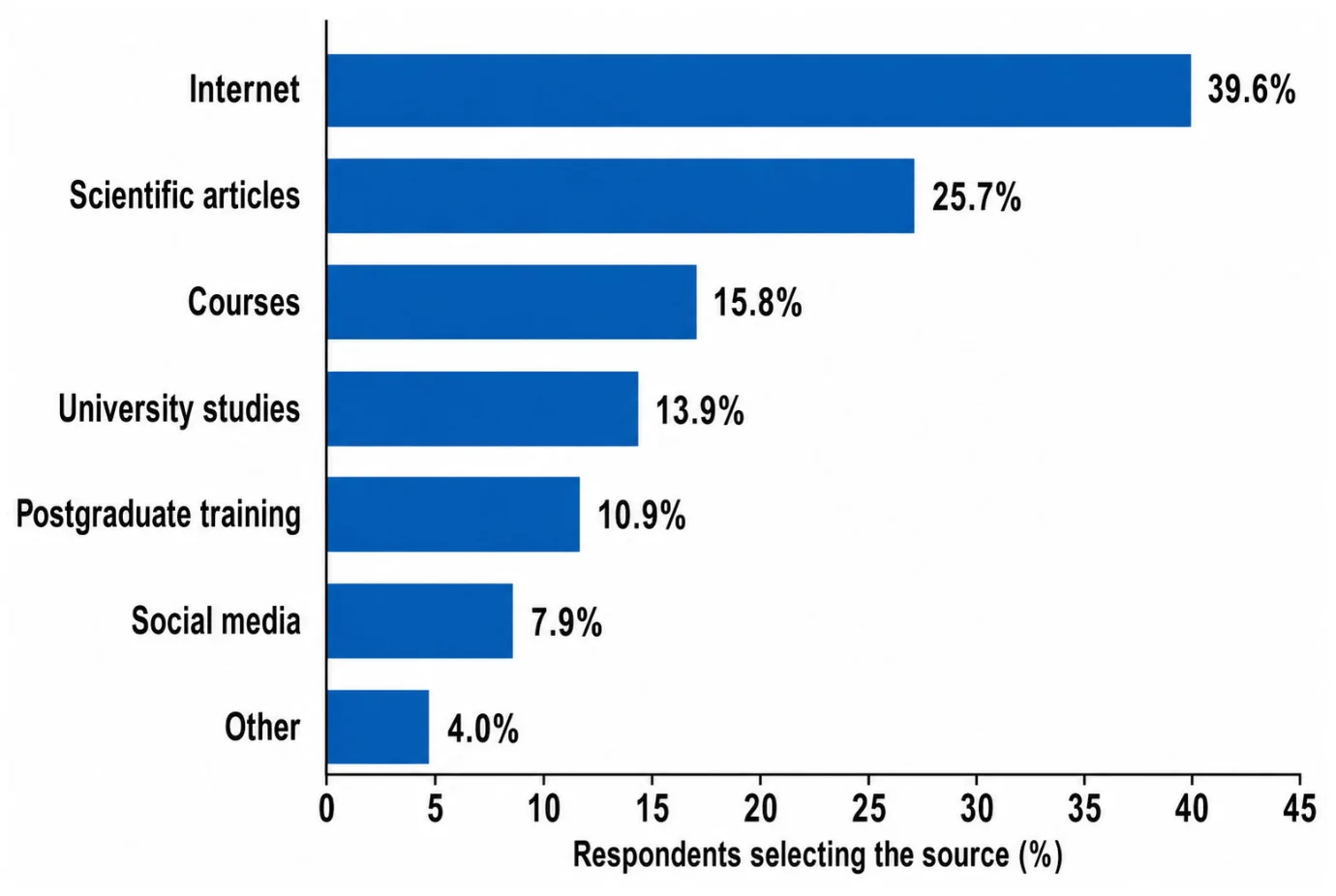
Main sources of self-rated knowledge about SWE and MyotonPro.

Age showed weaker positive associations, ranging from ρ = 0.24 to 0.40. After Holm adjustment, statistically significant associations remained for SWE self-rated knowledge (ρ = 0.40, adjusted *p* = 0.0003), MyotonPro self-rated knowledge (ρ = 0.29, adjusted *p* = 0.0183), SWE early-change beliefs (ρ = 0.39, adjusted *p* = 0.0009), and perceived access/cost barriers (ρ = 0.34, adjusted *p* = 0.0058). The associations of age with MyotonPro early-change beliefs, perceived combined diagnostic value, training willingness, MyotonPro therapy utility, and SWE monitoring utility did not remain statistically significant after multiplicity adjustment (all adjusted *p* = 0.0518). Overall, the correlation analyses demonstrated a more consistent relationship between professional experience and technology-related outcomes than between age and these outcomes (Table 4). The reported sources of knowledge about SWE and MyotonPro are shown in Figure 3.

The Internet was the most frequently reported source of information (39.6%), followed by scientific articles (25.7%), courses (15.8%), university studies (13.9%), postgraduate training (10.9%), social media (7.9%), and other sources (4.0%). Multiple sources could be selected; therefore, the percentages represent the proportion of respondents selecting each source and were not expected to sum to 100%.

Taken together, the findings demonstrate that awareness of SWE and MyotonPro was substantially higher than actual previous use, while access to either technology was reported by fewer than one-third of respondents. Practicing physiotherapists consistently reported greater awareness, access, previous use, knowledge, perceived utility, and recognition of implementation barriers than final-year students. Professional experience showed positive associations across all nine technology-related outcomes, whereas age demonstrated fewer and generally weaker associations. The findings also showed relatively high willingness to undertake training alongside limited self-rated knowledge and relatively low previous use.

## 4. Discussion

The present study identified a clear difference between final-year physiotherapy students and practicing physiotherapists in awareness, access, previous use, self-rated knowledge, and perceived clinical utility of SWE and MyotonPro. Practicing physiotherapists were substantially more likely to have previously encountered both technologies, to have access to them, and to have used them. The largest between-group differences concerned self-rated knowledge, with the largest rank-biserial effect sizes observed for SWE (r_rb = 0.618) and MyotonPro (r_rb = 0.509). These findings are consistent with greater familiarity with SWE and MyotonPro among practicing physiotherapists, but the observed differences cannot be attributed specifically to professional exposure or the transition from undergraduate education to clinical practice. The two groups also differed in age, professional experience, access to equipment, educational and workplace context, and potentially other unmeasured factors. Because the present study was cross-sectional and relied primarily on bivariate comparisons, professional experience should therefore be considered an associated factor rather than a causal determinant of self-rated knowledge or adoption.

The relatively limited use observed in the present study can be considered in the context of previous research on ultrasound imaging education and implementation in physiotherapy, although such evidence is not directly specific to SWE. In an international survey of 1307 physiotherapists from 49 countries, Ellis et al. [11] reported that 38% of respondents used ultrasound imaging (USI), whereas 62% were non-users. Among non-users, lack of training was the most frequently reported barrier (75.9%), followed by lack of equipment (55.5%) and equipment cost (42.4%). Importantly, USI users were older, had more clinical experience, were more likely to be research active, and more likely to hold postgraduate qualifications. This pattern is notably similar to our findings, in which professional experience showed positive associations with all nine technology-related outcomes (ρ = 0.30–0.62), with the strongest association observed for SWE self-rated knowledge (ρ = 0.62). Thus, the observed association between professional experience and greater familiarity may have several possible explanations, including exposure to these technologies in professional settings; however, the present study did not directly measure the frequency or duration of such exposure. The discrepancy between awareness and actual use is particularly important. In the present sample, only 15.8% of respondents had ever used SWE and 21.8% had ever used MyotonPro, while regular use was uncommon. These findings describe limited previous use in the surveyed population but do not establish what level of use would be clinically appropriate for either technology. A similar implementation context was reported by Ockerman et al. [13], who surveyed 305 physiotherapists in Flanders and found that 36.7% reported using no rehabilitation technology in clinical practice. Although attitudes toward technology were generally positive, insufficient knowledge, finances, time, and skills were identified as barriers. These findings provide relevant background regarding barriers that may be shared across physiotherapy technologies, but they should not be interpreted as evidence that the same barriers or their relative importance apply specifically to SWE and MyotonPro.

The implementation considerations may nevertheless differ between SWE and MyotonPro. SWE requires ultrasound equipment and appropriate training in image acquisition and interpretation, whereas MyotonPro is a surface-applied device with different measurement requirements and tissue-access limitations. Accordingly, equipment availability and ultrasound-specific training may be particularly relevant to SWE, while device-specific measurement procedures, parameter selection, and interpretation may be more relevant to MyotonPro. The present study did not assess the relative importance of these technology-specific factors, and therefore these distinctions should be considered as contextual considerations rather than empirically established determinants of use.

The strong association between professional experience and technology-related outcomes provides a context for considering educational factors, although the present study did not assess the adequacy or content of physiotherapy education. Savage et al. [12] reported that 70% of entry-level Doctor of Physical Therapy programs in the United States included USI education, but 91% devoted fewer than 10 h to this content. The principal barriers to expansion were lack of qualified instructors, insufficient knowledge or training, and equipment cost. Moreover, only half of respondents considered USI performance and interpretation to be an entry-level skill. These findings provide a relevant educational context for the markedly lower self-rated knowledge observed among students in the present study. However, the present study did not assess the adequacy of physiotherapy curricula or determine whether differences in self-rated knowledge were attributable to differences in formal education. Greater curricular exposure to ultrasound-based technologies may therefore represent a potential educational approach, but its necessity and effectiveness were not evaluated in the present study. The importance of structured training is further supported by evidence from physical medicine and rehabilitation. Bockbrader et al. [14] found that 80% of surveyed US physical medicine and rehabilitation residency programs taught musculoskeletal ultrasonography, whereas only 20% required competency for graduation. Major barriers included insufficient instructor expertise, limited equipment access, and lack of a structured curriculum. Together with the findings of Savage et al. [12] and Bockbrader et al. [14], these studies provide contextual support for considering competency-based education as a potential approach to ultrasound-related technology adoption. However, these findings do not establish that structured education is necessary for, or would improve, the adoption or competence of SWE or MyotonPro. This possibility should therefore be considered a hypothesis for future research rather than a conclusion of the present study. Overall, the available evidence supports the relevance of education, training, equipment access, and standardization as contextual implementation considerations, but direct evidence linking these factors to adoption or clinical competence for SWE and particularly MyotonPro among physiotherapists remains insufficient. The present findings should nevertheless be interpreted cautiously with regard to the perceived diagnostic potential of SWE. The concentration of responses at the lowest category for several perception items should also be interpreted cautiously. Because many respondents had not previously heard of SWE or MyotonPro, a low score may reflect limited familiarity with the technologies rather than a well-informed negative assessment of their potential utility. This interpretation is supported by the exploratory analyses, in which respondents with prior awareness or previous use reported higher median perception and utility scores than those without prior awareness or previous use. However, because these analyses were exploratory and based on relatively small subgroups, they should not be interpreted as evidence of a causal effect of prior experience on perceptions. Practicing physiotherapists were more likely than students to believe that SWE could identify early tissue changes and reported greater perceived clinical utility. However, these perceptions should not be equated with established diagnostic effectiveness. Borms et al. [4] recently reviewed the measurement properties of SWE for assessing rotator cuff muscle stiffness and included 42 studies. Their review found moderate evidence for positive intra- and inter-rater reliability for several rotator cuff muscles, but evidence regarding convergent validity, discriminant validity, and responsiveness was inconsistent. The authors therefore concluded that SWE is potentially valuable, but that further high-quality methodological research is needed to clarify its applicability in clinical practice and to improve the standardization of measurement procedures. Thus, the relatively strong belief in the potential of SWE observed among practicing physiotherapists may have several possible explanations, including greater familiarity with the technology; however, the present study did not directly measure the extent of prior exposure or familiarity in terms of frequency or duration of use. These perceptions should therefore not be interpreted as evidence of established diagnostic validity. A similar distinction is relevant to MyotonPro. The systematic review by Lettner et al. [6], which included 48 studies assessing 31 muscles, reported consistently high intra-rater and inter-rater reliability for several parameters, particularly frequency and stiffness. At the same time, the review identified substantial methodological heterogeneity, limited evidence for some parameters and anatomical sites, small samples in some studies, and insufficient longitudinal evidence. Therefore, the greater perceived therapeutic utility of MyotonPro among practicing physiotherapists in our study should be interpreted as a perception of potential clinical usefulness rather than evidence that the technology has established diagnostic or treatment-monitoring effectiveness. Importantly, direct evidence on education, professional adoption, and implementation of MyotonPro among physiotherapists remains limited; the available literature is predominantly concerned with measurement reliability, feasibility, and potential clinical applications rather than training or routine clinical uptake [15].

An important finding was the relatively high willingness to undertake training, despite low self-rated knowledge and limited previous use. This finding indicates an expressed interest in training among respondents, but does not establish that additional training is necessary or that it would increase clinical adoption. At the same time, the greater recognition of access and cost barriers among practicing physiotherapists indicates that these factors may be relevant to implementation, although their actual impact on clinical adoption was not evaluated. Previous physiotherapy surveys similarly identified equipment availability, cost, training, and lack of confidence as important barriers to implementation [11]. Education, equipment access, and organizational support may represent relevant areas for further investigation; however, the present study does not establish that any of these factors are responsible for the observed level of technology use, nor does it evaluate whether interventions targeting these factors would improve clinical adoption or patient outcomes. The predominance of the Internet (39.6%) and scientific articles (25.7%) as reported information sources should be interpreted descriptively. These findings do not demonstrate that respondents require structured education, nor do they establish that the type of information source affects competence or technology adoption. They may nevertheless support the hypothesis that future studies should examine whether structured, supervised education provides benefits beyond self-directed access to online resources and scientific literature. This is particularly relevant for SWE, for which measurement properties may vary depending on the protocol and technical conditions, and for MyotonPro, for which evidence remains heterogeneous across muscles and measurement parameters. A structured educational pathway could therefore focus on the principles of each technology, standardized measurement procedures, sources of measurement variability, interpretation of quantitative results, and integration with conventional clinical assessment rather than presenting either technology as a standalone diagnostic tool. Importantly, the present study did not compare SWE or MyotonPro with manual assessment or conventional functional measures and therefore cannot establish incremental clinical value or superiority over established approaches.

## 5. Limitations of the Study

Several limitations should be considered. The cross-sectional design does not permit causal inference. In addition, the use of voluntary purposive online sampling may have introduced volunteer and self-selection bias, as individuals with greater interest in emerging quantitative tissue-assessment technologies may have been more likely to participate. Recruitment within a single city (Warsaw, Poland) also limits the representativeness and generalizability of the findings. Because the specific recruitment groups, associations, and their membership sizes were not recorded, the representativeness of the recruitment sources and the potential for source-specific selection bias could not be fully assessed, which may limit the external validity of the findings. Although participants were recruited from different higher-education institutions and professional workplaces, all participants were studying or practicing in Warsaw. Therefore, the findings may not be generalizable to physiotherapy students and practicing physiotherapists in other regions of Poland or in different healthcare and educational contexts. The findings should likewise not be extrapolated to physiotherapists in other European countries, where educational systems, professional roles, healthcare structures, and access to these technologies may differ. The student subgroup consisted exclusively of final-year physiotherapy students, which may further limit the applicability of the findings to students at earlier stages of physiotherapy education. Because the number of individuals invited to participate and the corresponding response rate were not available, the extent of non-response bias could not be assessed. Most outcomes were self-reported and may therefore be affected by recall and response bias. The questionnaire did not provide a separate “unable to judge” or “do not know” response option for the ordinal perception items, and no substantive introductory information about SWE or MyotonPro was provided before these items. Consequently, low scores may partly reflect limited familiarity with the technologies rather than fully informed negative perceptions. The study did not assess clinical outcomes, incremental diagnostic or treatment-monitoring value, resource use, or cost-effectiveness of SWE and MyotonPro compared with established clinical assessment approaches. In addition, the questionnaire was developed specifically for this study and no formal psychometric validation was documented. Consequently, findings concerning knowledge, attitudes, and perceived clinical utility should be interpreted as study-specific self-reported indicators rather than validated measures of the corresponding underlying constructs; the knowledge findings in particular represent perceived rather than objectively demonstrated competence. Accordingly, the between-group differences should be interpreted as differences in reported responses to the study-specific items rather than definitive differences in objectively measured knowledge, underlying attitudes, or actual clinical utility. Because expert review, pilot testing, and item comprehensibility assessment were not performed, differential interpretation of some items between students and practicing physiotherapists cannot be excluded. Professional status and professional experience were inherently related in this sample, and the analytical approach was primarily based on bivariate comparisons and correlations; therefore, differences between students and practicing physiotherapists cannot be clearly disentangled from the effect of professional experience. The lack of multivariable modelling also means that the independent contributions of age, professional experience, and workplace setting could not be established. No a priori sample-size calculation was performed because recruitment was voluntary and constrained to the predefined data-collection period; the post hoc adequacy assessment was based on an observed effect and should not be interpreted as evidence of prospective power for smaller or clinically relevant effects. The relatively small sample may also limit the precision of subgroup estimates and the stability of estimates for individual outcomes.

Overall, the findings indicate that SWE and MyotonPro remain incompletely integrated into physiotherapy education and clinical practice despite greater awareness and more favorable perceptions among practicing physiotherapists. Professional experience was the most consistent correlate of technology-related outcomes, whereas limited access, cost, knowledge, and skills appear to represent potentially modifiable barriers. The findings indicate potential interest in structured and competency-oriented education regarding these technologies, but they do not establish that such education is necessary or would improve clinical adoption. At the same time, the current evidence base does not justify interpreting increased awareness or perceived utility as evidence of established diagnostic effectiveness. Further studies should therefore combine objective assessment of user competence with standardized measurement protocols and prospective evaluation of clinical validity and usefulness.

## 6. Conclusions

SWE and MyotonPro remain incompletely integrated into the surveyed physiotherapy population, despite greater awareness, self-rated knowledge, and perceived clinical utility among practicing physiotherapists than among students. Professional experience was the most consistent correlate of technology-related outcomes, while limited equipment access and cost represented important barriers to implementation. The findings identify reported interest in training as an area warranting further investigation. Whether structured, competency-oriented education would improve competence or clinical adoption of SWE or MyotonPro remains uncertain and requires direct evaluation. Given the cross-sectional design and the use of study-specific, non-validated self-report indicators, the findings should be interpreted as differences in reported awareness, self-rated knowledge, and perceived utility rather than definitive differences in objectively measured knowledge, underlying attitudes, or clinical effectiveness. Further research using standardized protocols, objective measures of competence, and appropriate clinical outcomes is needed to establish the clinical validity and added clinical value of SWE and MyotonPro and to determine whether broader implementation or procurement is justified.

## Figures and Tables

**Table 1:** Sociodemographic and professional characteristics of participants according to study group.

Characteristic	Students (n = 45)	Practicing Physiotherapists (n = 56)
Age		
<25 years	35 (77.8%)	7 (12.5%)
25–34 years	9 (20.0%)	36 (64.3%)
35–45 years	0 (0.0%)	12 (21.4%)
>45 years	1 (2.2%)	1 (1.8%)
Sex		
Female	20 (44.4%)	19 (33.9%)
Male	25 (55.6%)	37 (66.1%)
Educational level		
Bachelor’s degree	1 (2.2%)	1 (1.8%)
Master’s degree	1 (2.2%)	38 (67.9%)
Postgraduate education/specialization	1 (2.2%)	10 (17.9%)
Professional experience as a physiotherapist		
0–2 years	43 (95.6%)	18 (32.1%)
3–5 years	2 (4.4%)	27 (48.2%)
6–10 years	0 (0.0%)	9 (16.1%)
11–20 years	0 (0.0%)	1 (1.8%)
>20 years	0 (0.0%)	1 (1.8%)
Main work setting		
Rehabilitation center	—	25 (44.6%)
Hospital	—	19 (33.9%)
Private clinic	—	10 (17.9%)
Other	—	2 (3.6%)

Note: Values are presented as n (%). Study groups were defined according to participants’ main workplace. Educational level and professional experience were recorded and analyzed as separate characteristics.

**Table 2:** Awareness, access and self-reported use of SWE and MyotonPro according to professional status.

Outcome	Students, n (%)	Practicing, n (%)	χ^2^	*p*	φ
Prior awareness of SWE	3 (6.7%)	29 (51.8%)	23.47	<0.001	0.48
Prior awareness of MyotonPro	6 (13.3%)	30 (53.6%)	17.61	<0.001	0.42
Access to SWE or MyotonPro	4 (8.9%)	26 (46.4%)	16.84	<0.001	0.41
Ever used SWE	0 (0.0%)	16 (28.6%)	15.28	<0.001	0.39
Ever used MyotonPro	2 (4.4%)	20 (35.7%)	14.32	<0.001	0.38

Note: Values are presented as n (%). χ^2^, Pearson’s chi-square statistic; φ, phi coefficient. *p* values are two-sided.

**Table 3:** Between-group differences in self-rated knowledge, perceptions, training willingness, barriers and perceived clinical utility.

Outcome	Students, Median [IQR]	Practicing, Median [IQR]	U	Holm-Adjusted *p*	Rank-Biserial Correlation (r_rb)
SWE self-rated knowledge	0.00 [0.00–0.00]	2.00 [0.00–4.00]	452.0	<0.0001	0.618
MyotonPro self-rated knowledge	0.00 [0.00–0.00]	2.00 [0.00–4.00]	605.0	<0.0001	0.509
SWE early-change belief	1.00 [1.00–1.00]	3.00 [1.00–4.00]	519.5	<0.0001	0.513
MyotonPro early-change belief	1.00 [1.00–1.00]	3.00 [1.00–3.00]	642.5	0.0006	0.423
Combined diagnostic value	1.00 [1.00–1.00]	3.00 [1.00–3.00]	698.5	0.0022	0.369
Training willingness	1.50 [1.00–4.00]	4.00 [2.00–4.75]	842.0	0.0254	0.257
Access/cost barrier	1.00 [1.00–3.00]	4.00 [1.00–5.00]	640.0	0.0011	0.422
MyotonPro therapy utility	1.00 [1.00–2.00]	3.00 [1.00–3.00]	659.0	0.0014	0.397
SWE monitoring utility	1.00 [1.00–1.00]	3.00 [1.00–3.00]	667.5	0.0022	0.374

Note: Values are presented as median [IQR]. U, Mann–Whitney U statistic; *p* values are Holm-adjusted; r_rb, rank-biserial correlation. Self-rated knowledge items were scored on a 0–5 scale; all other outcomes were scored on a 1–5 ordinal scale.

**Table 4:** Professional experience and age in relation to technology-related outcomes.

Outcome	Age ρ	Age Holm *p*	Experience ρ	Experience Holm *p*
SWE self-rated knowledge	0.40	0.0003	0.62	<0.0001
MyotonPro self-rated knowledge	0.29	0.0183	0.42	<0.0001
SWE early-change belief	0.39	0.0009	0.48	<0.0001
MyotonPro early-change belief	0.26	0.0518	0.46	<0.0001
Combined diagnostic value	0.24	0.0518	0.42	0.0001
Training willingness	0.26	0.0518	0.30	0.0032
Access/cost barrier	0.34	0.0058	0.47	<0.0001
MyotonPro therapy utility	0.26	0.0518	0.35	0.0013
SWE monitoring utility	0.25	0.0518	0.42	0.0001

Note: ρ, Spearman’s rank correlation coefficient; *p* values are Holm-adjusted. All reported *p* values are two-sided.

## Data Availability

The dataset generated and analyzed during the current study is not publicly available due to privacy and confidentiality considerations related to the individual-level survey responses. Although the survey was anonymous and no direct personal identifiers were collected, the dataset contains detailed demographic and professional information that, particularly in combination, could potentially increase the risk of identification of individual participants. Therefore, the raw individual-level dataset is not publicly available. A suitably anonymized dataset or relevant data underlying the findings may be made available by the corresponding author upon reasonable request, subject to appropriate privacy and ethical considerations.

## Supplementary Materials

The following supporting information can be downloaded at: https://www.mdpi.com/article/10.3390/healthcare14203440/s1, Supplementary Material S1. Reconstructed Questionnaire Used in the Study. Supplementary Table S1. Exploratory analysis of technology-related perceptions according to prior awareness and previous use.

## Abbreviations

SWE	shear wave elastography
MRI	magnetic resonance imaging
IQR	interquartile range
