# Supplementary material for: Awareness, Access and Use of Shear Wave Elastography and MyotonPRO Among Physiotherapy Students and Practicing Physiotherapists: A Cross-Sectional Survey
Source: Healthcare (Basel). 2026 Oct 14;14(20):3440. doi: 10.3390/healthcare14203440 (PMC13704028; doi:10.3390/healthcare14203440)
Supplement: Supplementary file 1 [file healthcare-14-03440-s001.zip › healthcare-4593814-supplementary.pdf]

Supplementary Material S1.
Reconstructed Questionnaire Used in the Study.
Questionnaire: Physiotherapists' awareness of early overload-related changes in the musculoskeletal system using SWE and MyotonPro

The questionnaire presented below was reconstructed from the retained study documentation and response dataset. It includes the questionnaire items and response options available from the study records.

Introductory page

The purpose of the study is to assess physiotherapists' knowledge, experience, and attitudes towards shear wave elastography (SWE) and myotonometry (MyotonPro) for detecting early overload-related changes in the musculoskeletal system that may not yet be visible on imaging examinations (X-ray, MRI, B-mode ultrasound).
Participation in the study is voluntary, anonymous, and free of charge. Completion of the questionnaire takes approximately 10–15 minutes. No data enabling the identification of participants are collected.

1. Informed consent

I consent to participate in the anonymous study concerning the use of SWE and MyotonPro in the diagnosis of musculoskeletal overload.
Yes
No

Questionnaire items

1. Age
Response options: Below 25 years; 25–34 years; 35–45 years; Above 45 years
2. Educational level
Response options: Student; Bachelor's degree; Master's degree; Postgraduate studies
3. Workplace location
Response options: City with more than 500,000 inhabitants; City with 200,000–500,000 inhabitants; City with 50,000–200,000 inhabitants; City with fewer than 50,000 inhabitants; Rural area
4. Sex
Response options: Male; Female
5. Professional experience as a physiotherapist (years)
Response options: 0–2; 3–5; 6–10; 11–20; >20
6. Main workplace
Response options: Not applicable – I am a student; Rehabilitation centre; Hospital; Private clinic; Other
7. Have you encountered patients with overload-related symptoms despite no abnormalities on X-ray, MRI, or B-mode ultrasound?
Response options: Yes; No
8. How often do you use X-ray/MRI/B-mode ultrasound findings when making therapeutic decisions?

Response options: Never; Rarely; Sometimes; Often; Always
9. Have you previously heard of shear wave elastography (SWE)?
Response options: Yes; No
10. Have you previously heard of myotonometry (MyotonPro)?
Response options: Yes; No
11. Do you collaborate with or have access to elastography (SWE) or myotonometry (MyotonPro) examinations?
Response options: SWE; MyotonPro; Both; No
12. How would you rate your knowledge of the operating principle of SWE? (0–5 scale)
Response options: 0; 1; 2; 3; 4; 5
The retained dataset confirms a 0–5 numerical scale. Verbal endpoint labels were not retained.
13. How would you rate your knowledge of the operating principle of MyotonPro? (0–5 scale)
Response options: 0; 1; 2; 3; 4; 5
The retained dataset confirms a 0–5 numerical scale. Verbal endpoint labels were not retained.
14. What are your sources of knowledge about these techniques?
Response options: University studies; Courses; Postgraduate training; Scientific articles; Internet; Social media; Other; Not applicable
Multiple responses could be selected.
15. Have you used an SWE device in research or clinical practice?
Response options: Yes – regularly; Yes – occasionally; No, but I have access to the results; No
16. Have you used MyotonPro (myotonometer)?
Response options: Yes – regularly; Yes – occasionally; No, but I have access to the results; No
17. If yes, for which indications have you used MyotonPro?
Response options: Assessment of muscle tone (e.g. spasticity); Assessment of muscle condition after injury; Side-to-side comparison (L/R); Monitoring rehabilitation; Other; Not applicable – I have not used this method
Multiple responses could be selected.
18. If yes, for which indications have you used SWE?
Response options: Assessment of muscles; Assessment of tendon stiffness; Mapping areas of altered elasticity; Monitoring healing; Other; Not applicable – I have not used this method
Multiple responses could be selected.
19. SWE may detect early overload-related changes that are not visible on X-ray/MRI/B-mode ultrasound.
Response options: 1; 2; 3; 4; 5
Five-point numerical response scale. Verbal response labels were not retained.
20. MyotonPro (myotonometry) may detect early functional changes (tone, stiffness) before changes become visible on imaging.
Response options: 1; 2; 3; 4; 5
Five-point numerical response scale. Verbal response labels were not retained.

21. The combination of SWE + MyotonPro provides substantially more diagnostic information than either method alone.
Response options: 1; 2; 3; 4; 5
Five-point numerical response scale. Verbal response labels were not retained.
22. I would like to participate in training on the use of SWE and MyotonPro and interpretation of their results.
Response options: 1; 2; 3; 4; 5
Five-point numerical response scale. Verbal response labels were not retained.
23. Lack of equipment access and costs are the main barriers to implementing these methods in my workplace.
Response options: 1; 2; 3; 4; 5
Five-point numerical response scale. Verbal response labels were not retained.
24. Clinical scenario: A patient has Achilles tendon pain; MRI and B-mode ultrasound show no significant abnormalities, and symptoms have been present for 6 weeks. What would you consider?
Response options: Referral for SWE; MyotonPro measurement; Continue rehabilitation without additional testing; Other; Not applicable
Multiple responses could be selected.
25. Which MyotonPro parameters would be most useful to you?
Response options: Tone (oscillation frequency); Stiffness (N/m); Decrement (damping); Side-to-side comparison; Not applicable – I have not used this method
Multiple responses could be selected.
26. Which SWE findings would be most informative to you?
Response options: Wave velocity; Stiffness mapping in the region of interest (ROI); Comparison with the contralateral side; Monitoring changes over time; Not applicable – I have not used this method
Multiple responses could be selected.
27. Do you think SWE and MyotonPro findings should be confirmed using another method (e.g. power-Doppler ultrasound, MRI, functional tests)?
Response options: Yes; No; Not applicable – I have not used this method
28. How useful do you consider MyotonPro for assessing the effects of manual therapy (pre/post)?
Response options: 1; 2; 3; 4; 5
Five-point numerical response scale. Verbal response labels were not retained.
29. How useful do you consider SWE for monitoring treatment effects (e.g. shock-wave therapy, physical modalities)?
Response options: 1; 2; 3; 4; 5
Five-point numerical response scale. Verbal response labels were not retained.
30. What do you consider to be the greatest barriers to implementing SWE and MyotonPro in everyday physiotherapy practice?
Open-ended response.
31. What benefits do you see from using both methods in the assessment of overload-related conditions?
Open-ended response.

32. Methodological comments / suggestions regarding measurement protocols (if you have experience)
Open-ended response.

Supplementary Table S1. Exploratory analysis of technology-related perceptions according to prior awareness and previous use.

Outcome	Experience group	n	Median (IQR)
SWE early-change detection	Prior awareness of SWE	32	3 (2.75–4)
	No prior awareness of SWE	61	1 (1–1)
MyotonPro early-change detection	Prior awareness of MyotonPro	35	3 (3–4)
	No prior awareness of MyotonPro	60	1 (1–1)
SWE monitoring utility	Previous SWE use	16	4 (3–4)
	No previous SWE use	77	1 (1–3)
MyotonPro therapy utility	Previous MyotonPro use	22	3 (3–4)
	No previous MyotonPro use	72	1 (1–2)

Note: Values are presented as median (IQR) based on available valid responses. The analyses were exploratory and descriptive and were not intended to establish causal relationships.
